# Age-adjusted Charlson comorbidity index score as predictor of survival of patients with digestive system cancer who have undergone surgical resection

**DOI:** 10.18632/oncotarget.18401

**Published:** 2017-06-07

**Authors:** Yaohua Tian, Zhong Jian, Beibei Xu, Hui Liu

**Affiliations:** ^1^ Department of Epidemiology and Biostatistics, School of Public Health, Peking University, 100083 Beijing, China; ^2^ Hospital Administration Department, Peking University, 100083 Beijing, China; ^3^ Medical Informatics Center, Peking University, 100083 Beijing, China; ^4^ National Healthcare Data Center, Affiliated to National Center for Medical Service Administration, 100083 Beijing, China

**Keywords:** digestive system cancer, surgical resection, comorbidity, postoperative mortality, surgery

## Abstract

Comorbidities have considerable effects on survival outcomes. The primary objective of this retrospective study was to examine the association between age-adjusted Charlson comorbidity index (ACCI) score and postoperative in-hospital mortality in patients with digestive system cancer who have undergone surgical resection of their cancers. Using electronic hospitalization summary reports, we identified 315,464 patients who had undergone surgery for digestive system cancer in top-rank (Grade 3A) hospitals in China between 2013 and 2015. The Cox proportional hazard regression model was applied to evaluate the effect of ACCI score on postoperative mortality, with adjustments for sex, type of resection, anesthesia methods, and caseload of each healthcare institution. The postoperative in-hospital mortality rate in the study cohort was 1.2% (3,631/315,464). ACCI score had a positive graded association with the risk of postoperative in-hospital mortality for all cancer subtypes. The adjusted HRs for postoperative in-hospital mortality scores ≥ 6 for esophagus, stomach, colorectum, pancreas, and liver and gallbladder cancer were 2.05 (95% CI: 1.45–2.92), 2.00 (95% CI: 1.60–2.49), 2.54 (95% CI: 2.02–3.21), 2.58 (95% CI: 1.68–3.97), and 4.57 (95% CI: 3.37–6.20), respectively, compared to scores of 0–1. These findings suggested that a high ACCI score is an independent predictor of postoperative in-hospital mortality in Chinese patients with digestive system cancer who have undergone surgical resection.

## INTRODUCTION

Digestive system cancer, including those in the digestive tract and accessory organs, has the highest incidence and mortality of all cancers. In the USA, an estimated 310,440 new cases and 157,700 deaths from digestive system cancer are expected to occur in 2017 [1]. According to 2015 cancer statistics for China, stomach, esophageal, and liver cancers were the commonest cancers and the leading causes of cancer-related death [2]. Despite advances in screening programs and chemoradiation treatment over the last few decades, surgical resection remains the mainstay of curative treatment for patients presenting with digestive system cancer. It has been estimated that more than 250,000 patients undergo major cancer surgery in the USA annually [3]. The prognoses of patients after cancer surgery have aroused increasingly more attention [4–6]. Despite a steady decline attributable to continuous improvements in perioperative care, service and management ability and surgical techniques, postoperative mortality remains one of the most feared postoperative complications. A prospective multicenter cohort study across 28 European nations reported that in-hospital postoperative mortality rate was as high as 4% [7]. Preoperative stratification of patients has important clinical implications in individual decision-making, treatment selection, and postoperative care.

Several studies have suggested that a high prevalence of medical comorbidities in patients with digestive system cancer [8, 9] significantly impact screening strategies [10], therapeutic planning [11], and prognosis [12]. Hence, comorbidities should be adjusted for as confounding variables in health outcome studies. Several statistical methods have been developed to measure and assess the overall burden of comorbidities [13]. Of these, the Charlson comorbidity index (CCI), which comprises 19 weighted comorbidity items [14] and has been validated in various clinical settings, is widely used [15–17]. Previous studies have demonstrated that digestive system cancer patients with higher burden of comorbidities as measured by CCI score have significantly poorer postoperative short-term and long-term survival rates [18–20] In addition, age is reportedly a significant predictor of survival outcome and has therefore been incorporated into the CCI score to create a single index that accounts for both comorbidity and age, the age-adjusted Charlson comorbidity index (ACCI) [18]. However, previous studies on this comorbidity index have mostly been carried out in Western countries. To our knowledge, there has never been a large multicenter study in China aimed at evaluating the effects of comorbidities measured by a validated comorbidity score on postoperative mortality of patients with digestive system cancer. Because of the considerable differences in patterns of comorbidities among populations in different regions of the world or races [19, 20], the influence of comorbidities on postoperative prognosis of Chinese patients is still unknown. With the continuous improvement of healthcare information systems in China [21], the quality of medical data has gradually improved, providing an opportunity to use massive amounts of health data to study comorbidities and their impacts on health outcomes in Chinese individuals.

The primary objective of this study, which was based on a multicenter national database, was to explore the risk of postoperative in-hospital mortality in patients with digestive system cancer who had undergone surgical resection and had varying comorbidity levels as measured by ACCI scores.

## RESULTS

Table 1 shows the baseline characteristics of patients who had undergone surgical resection of digestive system cancer from 2013 to 2015 by ACCI group. Of the 315,464 patients, 48,192 (15.3%) had low ACCI scores (0–1), 150,963 (47.9%) ACCI scores of 2–3, 74,818 (23.7%) had ACCI scores of 4–5, and 41,491 (9%) high ACCI scores (≥ 6). The mean ages (SD) of these four groups were 42.2 (7.6), 59.2 (6.6), 70.1 (8.7), and 64.7 (12.3) years, respectively. The proportions of sex, surgery type, and anesthesia method were inconsistent across the four ACCI groups (*P* < 0.001).

**Table 1:** Demographic characteristics of the digestive system cancer patients who underwent surgical resection from 2013 to 2015

Variable	Total	ACCI 0–1	ACCI 2–3	ACCI 4–5	ACCI ≥ 6	*P*-value
Total (%)	315464	48192 (15.3)	150963 (47.9)	74818 (23.7)	41491 (13.2)	
Age (mean ± SD)	59.9 ± 12.0	42.2 ± 7.6	59.2 ± 6.6	70.1 ± 8.7	64.7 ± 12.3	< 0.001
Sex						< 0.001
Men (%)	208382 (61.6)	29671 (61.6)	102614 (68.0)	48658 (65.0)	27439 (66.1)	
Women (%)	107082 (33.9)	18521 (38.4)	48439 (32.0)	26160 (35.0)	14052 (33.9)	
Surgery type						< 0.001
Open (%)	238163(75.5)	36886 (76.5)	116330 (77.1)	55397 (74.0)	29550 (71.2)	
Laparoscopic (%)	77301(24.5)	11306 (23.5)	34633 (22.9)	19421 (26.0)	11941 (28.8)	
Anesthesia method						< 0.001
General (%)	280508 (88.9)	42835 (88.9)	135403 (89.7)	65979 (88.2)	36291 (87.5)	
Regional (%)	34956 (11.1)	5357 (11.1)	15560 (10.3)	8839 (11.8)	5200 (12.5)	

ACCI: age-adjusted Charlson comorbidity index.

Table 2 presents postoperative in-hospital mortality according to ACCI categories. The postoperative in-hospital mortality rate increased steadily across ACCI groups, ranging from 0.6% among patients with ACCI score of 0–1 to 0.9% among patients with ACCI score of 2–3, 1.6% among patients with ACCI score of 4–5, and 2.0% among patients with ACCI score ≥ 6. A trend toward increased postoperative in-hospital mortality rate with higher ACCI score was consistently noted for all cancer subtypes.

**Table 2:** Mortality among digestive system cancer patients who underwent surgical resection by cancer types and age-adjusted Charlson comorbidity index score from 2013 to 2015

Cancer type	Total	Event (%)	*P*-value
Overall	315464	3631 (1.2)	< 0.001
ACCI score 0–1	48192	270 (0.6)	
2–3	150693	1319 (0.9)	
4–5	74818	1199 (1.6)	
≥ 6	41491	843 (2.0)	
Esophagus	52495	798 (1.5)	< 0.001
ACCI score 0–1	4976	42 (0.8)	
2–3	30738	386 (1.3)	
4–5	11345	248 (2.2)	
≥ 6	5436	122 (2.2)	
Stomach	103799	1293 (1.2)	< 0.001
ACCI score 0–1	14788	113 (0.8)	
2–3	53329	511 (1.0)	
4–5	23047	399 (1.7)	
≥ 6	12635	270 (2.1)	
Colorectum	124246	1123 (0.9)	< 0.001
ACCI score 0–1	18921	91 (0.5)	
2–3	52466	286 (0.5)	
4–5	32397	406 (1.3)	
≥ 6	20462	340 (1.7)	
Liver and gallbladder	68343	842 (1.2)	< 0.001
ACCI score 0–1	11964	51 (0.4)	
2–3	31414	297 (0.9)	
4–5	15511	266 (1.7)	
≥ 6	9454	228 (2.4)	
Pancreas	18125	390 (2.2)	< 0.001
ACCI score 0–1	2884	29 (1.0)	
2–3	8948	159 (1.8)	
4–5	4063	127 (3.1)	
≥ 6	2230	75 (3.4)	

ACCI: age-adjusted Charlson comorbidity index.

Table 3 shows adjusted HRs for postoperative in-hospital mortality by cancer subtypes with ACCI scores. There was a positive graded association between postoperative in-hospital mortality and ACCI groups. For all cancer surgeries, patients with scores of 4–5 and ≥ 6 had significantly higher risk of postoperative mortality than those with ACCI scores of 0–1 after controlling for sex, type of resection, anesthesia methods, and caseload of each healthcare institution. For esophagus, stomach, colorectum, pancreas, and liver and gallbladder cancers, patients with ACCI scores ≥ 6 had 105% (HR, 2.05; 95% CI, 1.45–2.92), 100% (HR, 2.00; 95% CI, 1.60–2.49), 154% (HR, 2.54; 95% CI, 2.02–3.21), 158% (HR, 2.58; 95% CI, 1.68–3.97), and 357% (HR, 4.57; 95% CI, 3.37–6.20), respectively, greater risk of in-hospital death than those with ACCI scores of 0–1.

**Table 3:** Adjusted hazard ratios of individual age-adjusted Charlson comorbidity index score for postoperative in-hospital mortality

	Adjusted HR^a^	95% CI	*P*-value
Overall	1.2%		
ACCI score 0–1	1		
2–3	1.40	1.22–1.59	< 0.001
4–5	2.34	2.05–2.67	< 0.001
≥ 6	2.65	2.31–3.04	< 0.001
Esophagus	1.5%		
ACCI score 0–1	1		
2–3	1.33	0.97–1.83	0.080
4–5	2.07	1.49–2.87	< 0.001
≥ 6	2.05	1.45–2.92	< 0.001
Stomach	1.2%		
ACCI score 0–1	1		
2–3	1.08	0.88–1.32	0.481
4–5	1.63	1.32–2.01	< 0.001
≥ 6	2.00	1.60–2.49	< 0.001
Colorectum	0.9%		
ACCI score 0–1	1		
2–3	1.13	0.89–1.43	0.310
4–5	2.20	1.75–2.76	< 0.001
≥ 6	2.54	2.02–3.21	< 0.001
Liver and gallbladder	1.2%		
ACCI score 0–1	1		
2–3	2.15	1.60–2.89	< 0.001
4–5	3.64	2.70–4.91	< 0.001
≥ 6	4.57	3.37–6.20	< 0.001
Pancreas	2.2%		
ACCI score 0–1	1		
2–3	1.56	1.05–2.31	0.029
4–5	2.40	1.60–3.59	< 0.001
≥ 6	2.58	1.68–3.97	< 0.001

ACCI: age-adjusted Charlson comorbidity index. ^a^Adjust for sex, caseload of each healthcare institution, resection type of surgery, and anesthesia methods.

## DISCUSSION

In this study of data obtained from a national database in China on over 300,000 resection surgeries for digestive system cancer between 2013 and 2015, age and medical comorbidities as measured by ACCI scores had considerable impacts on the risk of postoperative in-hospital mortality. Patients with higher ACCI scores were at greater risk of death. To the best of our knowledge, this is so far the largest study worldwide to examine the association between comorbidity and postoperative mortality among patients with digestive system cancer. ACCI scores provide quantification of the prognostic effect of age and comorbidities on the risk of mortality after resection. With increasingly more attention being given to research aimed at improving the health outcomes of patients with cancer undergoing surgical resection [12, 22], the ACCI algorithm promises to be a highly useful tool for such studies.

In this study, the primary outcome was postoperative in-hospital mortality. Prognosis of patients with cancer who have undergone surgical resection has been poorly evaluated in China. To the best of our knowledge, the current study is the first to explore postoperative mortality in Chinese patients with cancer at a national level. The postoperative in-hospital mortality rate in the 172 top-ranked hospitals during 2013–2015 was 1.2%, which is broadly comparable with prior reports. A retrospective cohort study of 182,886 patients undergoing surgical interventions during 2006–2011 in a German tertiary care university hospital reported a postoperative in-hospital mortality rate of 1.3% [23]. Similarly, in another retrospective cohort study of 49 American College of Surgeons Commission on Cancer (CoC) accredited hospitals, an in-hospital mortality rate for inpatient cancer surgery of 2.1% was noted in 19 low mortality hospitals versus 9.1% in 30 high mortality hospitals [3]. Variations in postoperative in-hospital mortality within health-care systems may be partially attributable to type of hospitals [3, 7]. A major fact to note is that in this study the postoperative in-hospital mortality rate was for top-ranked hospitals in China.

Similar studies worldwide have shown that the presence of comorbidities is associated with a higher risk of postoperative mortality than the absence of comorbidities among patients with cancer. A prospective cohort study in the USA suggested a dose-response relationship between severity of comorbidity and survival among 17,712 cancer patients [12]. Similarly, a retrospective, single-institution analysis involving 497 patients who underwent surgery for pancreatic ductal adenocarcinoma between 2002 and 2012 found that patients with ACCI scores of ≥ 6 had a 189% higher risk of death within the first year than those with lower ACCI scores [24]. A recent study of data for 156,151 patients who underwent cancer surgery obtained from the Taiwan National Health Insurance Research Database demonstrated that patients with ACCI scores of 4–7, 9–11, and ≥ 12 had 2.84-fold (95% CI, 2.59–3.12), 6.07-fold (95% CI, 5.51–6.68), and 11.17-fold (95% CI, 9.97–12.50), respectively, higher risks of 90-day mortality than patients with scores of 0–3 [8]. ACCI scores have also been validated for other digestive and nondigestive cancers, specifically colorectal cancer [25], bladder cancer [26], early-stage endometrial cancer [27], and nasopharyngeal cancer [28]. The broad consistency across published studies indicates that the association between comorbidity and postoperative mortality among patients with cancer is unlikely to be substantially influenced by such characteristics as cancer type, geography, race, or study design.

The American Society of Anesthesiologists (ASA) physical status classification system is currently the most widely used preoperative risk measure for assessing the fitness of patients before surgery in routine clinical practice. However, possibly because of the subjective nature and poor inter–rater reliability of the ASA classification system [29], CCI scores have been demonstrated to be superior to ASA fitness scores for predicting postoperative mortality [23]. In addition, the effect of age is not taken into account in the ASA classification system, despite the potential effect of advanced age on postoperative mortality. Although malignant tumor occur at all ages, cancer disproportionately strikes individuals aged 65 years or older [30]. With the rapid aging of the population in China and many other countries [31], more and more older persons are expected to undergo cancer surgery. This study also illustrates the necessity to account for age, having found that advanced age is an independent predictor of postoperative mortality. Taken together, this evidence indicates the importance of weighing the impacts of age, such as medical comorbidities, on postoperative mortality risk. ACCI scores allow integration of both comorbidities and age in a single index and are thus more inclusive than either comorbidity or age alone.

Because of the substantial effects of age and comorbidity on screening strategies [10], treatment options [11], and survival outcome [12] among patients with cancer, ACCI scores with risk estimates may be of value in clinical practice. ACCI scores could be used as a single index of the overall burden of comorbidities and age in health outcome studies, significantly reducing the impact of confounding bias from these factors without necessitating the extremely large sample size that would be required to control for each condition separately [18]. Quantification of strong risk factors is important in controlling for confounding effects in health outcome studies using administrative databases [32]. The present study also illustrates the necessity to account for age and comorbidity. The postoperative in-hospital mortality rate in patients with ACCI scores of ≥ 6 was 233.3% higher than that of those with ACCI scores of 0–1. Prediction of postoperative mortality is important in perioperative risk assessment. ACCI scores may also be useful in identifying high- or low-risk subgroups, leading to more tailored approaches to patient management and possibly helping surgeons to determine the optimal treatment options for individual patients. In addition, using comorbidity scores as a screening tool would facilitate patients being cared for in suitable settings. ACCI scores should therefore be taken into account when planning individual treatment courses. Several studies have shown that comorbid conditions also have substantial influences on quality of life among cancer sur*vivo*rs and should be taken into consideration when managing follow-up care after primary treatment [33]. Future studies are warranted to validate these and other possible clinical applications of ACCI scores.

Unique features of this study include the large sample size, national multicenter design, and coverage of all main subtypes of digestive system cancer. One critical issue in this study is the data quality of the HSR data. All the 172 hospitals included in the study were reputable university-teaching hospitals, and enjoy the prestigious esteems for quality in all aspects of healthcare, including diagnosis, treatment, coding, hospital management, and electronic medical record systems in China. To fulfill the administrative requirements of the China Ministry of Health, every hospitalization in these hospitals must submit an electronic HSR to a centralized health information system for evaluating hospital performance. The Beijing Municipal Health Bureau had conducted a quality control study, and found that more than 95% of diagnostic codes recorded on the HSR were accurate based on manual examinations of electronic medical records [34]. Only inclusion of top-ranked hospitals can help with eliminating potential confounding bias caused by variations in surgical strategy, surgeons’ expertise, and hospital setting and quality, and focus on the role of age and comorbidities. However, the quality of care in other hospitals may not be as well as these top-ranked hospitals. Therefore, the generalizability of our results to other hospitals should be interpreted with caution.

Our study has a few noteworthy limitations. First, the retrospective data collection and design may have introduced some confounding bias. Second, although the validity and reliability of the ACCI for measurement of age and comorbidities has been verified, it is possible that some other comorbid conditions that may contribute to postoperative mortality, such as hypertension, are not included in the ACCI. In addition, with advances in medical care and chronic disease management, weighting algorithms developed in 1984 may no longer be applicable. However, the 19 comorbid conditions in the CCI capture the overwhelming majority of comorbidities encountered in patients with digestive system cancer and consistent significant associations between ACCI scores and postoperative mortality were observed across all cancer subtypes. These results confirm the validity of the ACCI in predicting the postoperative mortality of patients with digestive system cancer. Another limitation was our inability to account for cancer stage, histology, tumor burden, and other risk factors, such as smoking status and dietary habits, as such information is not in our database. However, given the robustness of the evidence, statistical analysis, and large sample size in this study, these limitations are unlikely to have compromised our findings.

In conclusion, our data demonstrate the significant impacts of age and comorbidity on overall survival in patients with digestive system cancer who have undergone surgical resection. Higher ACCI scores are associated with increased risk of postoperative mortality. ACCI scores may be helpful for preoperative risk assessment, treatment selection, and individual decision-making among patients with digestive system cancer.

## MATERIALS AND METHODS

### Data source

Data used in this study were obtained from electronic hospitalization summary reports (HSR) from top-rank (Grade 3A) hospitals that were being evaluated by the National Hospital Performance Project in the National Healthcare Data Center. The standard ranking system involves various factors, including hospital infrastructure, service and management ability, quality and safety of clinical care, and technical level and efficiency. The ranking system has three grades and ten classes, the highest rank being Grade 3A. In these hospitals, an electronic HSR for every hospitalization must be submitted to a centralized health information system as mandated by the administrators. Data recorded on the electronic HSR include basic patient variables (e.g., sex and age), dates of admission and discharge, hospitalization and discharge diagnoses and the corresponding International Classification of Diseases, 10th Revision (ICD-10) codes, surgical procedures and their corresponding International Classification of Diseases, 9th Revision, Clinical Modification (ICD-9-CM) codes, discharge status (survival status, hospitalization infection, and drug allergy), and financial costs.

An updated electronic HSR has been used since 2012. The updated HSR contains up to 11 listed ICD-10 coding discharge diagnoses and up to 10 ICD-9-CM coding fields for procedures. The first listed diagnosis is designed to record principle diagnosis or primary illness and other listed diagnoses are to capture comorbid conditions and complications. A new variable is assigned next to each listed diagnosis to specify the timing of diagnosis. Informed consent was not specifically obtained since the data used was collected for administrative purpose without personal identifiers.

In this study, patients presenting with esophagus (ICD-10 code: C15), stomach (ICD-10 code: C16), colorectum (ICD-10 code: C18–C21), pancreas (ICD-10 code: C25), or liver and gallbladder cancer (ICD-10 code: C22–C24) between 2013 and 2015 were identified from the HSR database. Next, we identified patients who had undergone a surgical procedure for one of the digestive system cancer types among these individuals with digestive system cancer. The surgical procedures for each cancer were identified using their ICD-9-CM codes. To minimize the effect of coding inaccuracy, we also used the corresponding Chinese terms to check the identified cases. Research indicated that natural language processing was an efficient method for identifying cases in large clinical databases [35, 36]. Patients were included in this study if they were aged 18 or above. Finally, 315,464 patients who had undergone a surgical procedure for one of the digestive system cancer types in 172 hospitals in 26 large cities across China were included in this study.

### Measurements

The primary outcome was in-hospital mortality, defined as any death occurring after surgical resection, in-hospital mortality reportedly being a more reliable quantifier than 30-day mortality [22]. The variables assessed were sex, age, type of resection, anesthesia methods, caseload of each healthcare institution, and comorbid conditions. ACCI scores were calculated using validated Charlson coding algorithms [14], in which 19 different medical categories are weighted based on their impacts on mortality with additional points added for age. In the ACCI, the age at which cancer surgery is performed is adjusted by adding one point for each decade after 40 years of age up to a total of four points (four points for ≥ 71 years). The weights and ICD-10 codes for the 19 comorbid conditions are listed in Table 4. The coding algorithms were carefully checked and reviewed by hospital management experts, epidemiologists, and physicians from our institution and reviewed again by several outside physicians. The ACCI score was calculated for each patient by identifying all comorbidities and summing the corresponding weights. The presence of comorbidities of each patient was identified from the ICD-10 codes recorded in the HSR. Comorbidities that were diagnosed after admission were excluded. The higher the ACCI score, the greater is the comorbid disease burden.

**Table 4:** ICD-10 coding algorithms and weights for comorbid conditions of age-adjusted Charlson comorbidity index

Comorbidity	weight	ICD-10
Myocardial infarction	1	I21 I22 I23 I24 I25
Congestive heart failure	1	I09.9 I11.0 I13.0 I13.2 I42 I43 I50 I51.7 I26
Peripheral vascular disease	1	I70-I74 I77 I79.0 I79.2 I80 K55.1 K55.8 K55.9 Z95.1 Z95.5 Z95.8 Z95.9
Cerebrovascular disease	1	G45 G46 H34.0 I60- I69
Dementia	1	F00-F03 F05.1 G30 G31.0 G31.1
Chronic pulmonary disease	1	I27.8 I27.9 J40-J47 J60-J67 J68.4 J70.1 J70.3 J84 J94 J96 J98
Connective tissue disease	1	L94.0 L94.1 L94.3 M05 M06 M08 M12.0 M12.3 M30-M36 M45 M46.1 M46.8 M46.9
Ulcer disease	1	K25-K28 K92
Mild liver disease	1	B18 K70.0-K70.3 K70.9 K71.3-K71.5 K71.7 K71.8 K73-K76 R94.5
Diabetes	1	E10.0 E10.1 E10.5 E10.6 E10.8 E10.9 E11.0 E11.1 E11.5 E11.6 E11.8 E11.9 E12.0 E12.1 E12.6 E12.8 E12.9 E13.0 E13.1 E13.5 E13.6 E13.8 E13.9 E14.0 E14.1 E14.5 E14.6 E14.8 E14.9
Hemiplegia	2	G04.1 G11.4 G80.1 G80.2 G81-G83
Moderate or severe renal disease	2	I12 I13.1 I13.9 I15 N00 N01 N03 N05 N07.2-N07.4 N13.3 N17-N20 N25 N28 N39 N40 Z49.0-Z49.2 Z94.0 Z99.2
Diabetes with end organ damage	2	E10.2-E10.4 E11.2-E11.4 E13.2-E13.4 E14.2-E14.4
Any solid tumor, leukemia and lymphoma	2	C00-C14 C30-C34 C37-C41 C43 C45-C58 C60-C76 C80-C86 C88 C90-C97 Z85
Moderate or severe liver disease	3	B15.0 B16.0 B16.2 B19.0 I85 I86.4 I98.2 K70.4 K71.1 K72
Metastatic solid tumor	6	C77-C79
AIDS	6	B20-B24
Age^a^	1	

AIDS: Acquired immunodeficiency syndrome. ^a^For each decade over age 40 years, up to 4.

### Statistical analysis

In this study, patients were categorized into four groups based on ACCI score: 0–1, 2–3, 4–5, and ≥ 6. Categorical variables are reported as percentages (%) and numerical data as mean ± standard deviation (SD). Differences were analyzed with the anova test for numerical variables and Pearson’s χ^2^ test for categorical variables. The Cox proportional hazard regression model was used to calculate hazard ratios (HRs) and 95% confidence intervals (95% CIs) across all ACCI groups. All analyses were conducted using the R programming language (V.3.2.2, R Development Core Team). All statistical tests were two-sided, and *P* < 0.05 was considered to denote statistical significance.
